# Supplementary material for: The effect of casein glycomacropeptide versus free synthetic amino acids for early treatment of phenylketonuria in a mice model
Source: PLoS One. 2022 Jan 11;17(1):e0261150. doi: 10.1371/journal.pone.0261150 (PMC8751992; doi:10.1371/journal.pone.0261150)
Supplement: Attachment — Submitted filename: Response to reviewers.rtf [file pone.0261150.s001.rtf]

Response to reviewers
(Reviewer #1) Thank you very much for your suggestion regarding using one-way ANOVA instead of multiple t tests.  During my PhD thesis, I did seek advice at the Statistical Department at the University of Copenhagen and the teacher recommended that I should use multiple t tests in order to obtain the most transparent results. He stated, that in his opinion it can confuse the outcome to include to many parameters in the same statistical analysis. Therefore, I choose to keep this method as kindly offered from the Academic Editor. 
As suggested, we have converted the bar graphs Figure 3 and supplementary Figure 1 to whisker plots. For Figure 3, we have separated the amino acids from the neurotransmitters into different charts to make the inspection of the current figure easier.
We do agree that a suitable short phrase or abbreviation would be preferable for naming the actual treatment of the groups. However, unfortunately, we have not been able to find a suitable better alternative for the current names.
Thank you for addressing this very important issue regarding lack of wild type mice. We did not have a wild type mouse to compare to, which we initial found was not a problem, since the main objective of the study was to determine the differences between the diet compositions. We are aware that similar studies has used wild type mouse to compare with ( (Ney, 2008) (Vliet, 2016) and we have realized afterwards, that this could have been useful for the Barnes maze study, since there were no differences between the groups, despite the different diet regimes. We have included a comment about this in the discussion section.
The experimental diet started at week 4, right after weaning was ended. The breeding animals were maintained on Phe-free semi synthetic diet (Harlan Laboratories), also during pregnancy and weaning period, a Maternal PKU diet. As the diet was free of Phe, the drinking water was supplemented with Phe (Sigma-Aldrich Chemie) to a final concentration of 62.5 mg/ml during that period. 
We are aware, that there are different opinions about whether the imbalance in brain LNAA is the primary cause of disrupted brain development in PKU, but instead of eliminate the sentence, we have altered the sentence and have added several other publications to document this statement.
Thank you for pointing out that statement about the potential pathogenesis of hyperphenylalaninemia upon bone health was missing. It has now been added.
Also thank you for emphasizing, that it is important to highlight the fact, that taking large amounts of large neutral amino acids orally can help protect the brain from damage from elevated phenylalanine, but the main lowering effect on brain phenylalanine comes from lowering the blood phenylalanine. We thought we already indicated that but have now expanded the discussion section where we describe in more detail with the work of others, as you advised us to do.
(Reviewer #2): Thank you for your valuable advices regarding design of the manuscript. We do agree that the text can be dry to read, and therefore we have combined “results-“and “discussion sections” a bit more as suggested and included the supplementary data in the main part of the manuscript. 
The composition of G3 is a complete diet, consisting of casein ( MIPRODAN^®^ 30), sucrose, corn starch, corn oil, cellulose, mineral- and vitamin-mix. Basically, the nutritional value is the same as the other experimental groups; the only difference is the protein source. MIPRODAN^®^ 30 Sodium Caseinate is a spray-dried pure milk protein manufactured from fresh pasteurized skimmed milk by acid precipitation of the casein, direct neutralization and spray-drying.
Thank you for pointing out that 57mg/ml of Phenylalanine supplementation would be an extraordinary amount of Phe (345 mM) and not suitable for the mouse strain.  In fact, 25 mg/ml was used to produce the drinking mixture, where 9 ml (225 mg) was added to 391 ml of water, which provided a final concentration of 563 mg/l 
Page 19/20: There are two error messages in the text. Thank you for making us aware of this, it is corrected now.
Table 2: Threonine is elevated in G4 and G5. It is an essential AA and diet 5 has a significant amount, but diet 4 doesn't seem to have Thr. Please explain. Arginine is consistent and found in G4 and G5 diets.
We are glad that you pointed this out, since it is not clear at all from the table 2, that the CGMP product contains large amount of Thr. We have now added the data from CGMP-20 to make it more obvious.
We hope the resolution of the figures have improved with the new system: uploading figure files to the Preflight Analysis and Conversion Engine (PACE) digital diagnostic tool  https://pacev2.apexcovantage.com/ 
Please present control data from a background mouse strain. Unfortunately, the mouse strain we used from AAU had no wild type as described in the introduction section, but we have found useful information from other similar studies (Ney, 2008) (Vliet, 2016)
Do not abbreviate brain areas. We are aware that this is a lot of abbreviations, but we find it necessary to leave as is in order to keep the same procedure through the entire manuscript regarding abbreviations.
Once again, we thank you for the time you put in reviewing our paper and look forward to meeting your expectations. Since your inputs have been precious, in the eventuality of a publication, we would like to acknowledge your contribution explicitly.
Sincerely yours,
Kirsten Kiær Ahring
Metabolic nutritionist, Ph.D.
Copenhagen University Hospital
Copenhagen, Denmark
