# Supplementary material for: The effect of casein glycomacropeptide versus free synthetic amino acids for early treatment of phenylketonuria in a mice model
Source: PLoS One. 2022 Jan 11;17(1):e0261150. doi: 10.1371/journal.pone.0261150 (PMC8751992; doi:10.1371/journal.pone.0261150)
Supplement: Attachment — Submitted filename: Response to reviewers.rtf [file pone.0261150.s002.rtf]

											April 24, 2021, Copenhagen, Denmark
PONE-D-19-32961
Dear Clemens Fürnsinn and reviewers,
September 1^st^, I re-submitted the manuscript with the title: “Effect of offering casein glycomacropeptide versus free synthetic amino acids to early treated phenylketonuria mice”.
I included a Cover letter (addressed to editor and reviewers) but also a “Response to reviewers” letter, which you find below. At October 8, I received a rejection of the manuscript and a note from the editor stating that “did not respond as expected”. However, I think there may have been a misunderstanding since in the letter below, we are addressing all the issues brought up by reviewer 1 and 2. I have now included the comments from the cover letter also and hope this will make the feedback more complete. PLOS ONE kindly allowed me to appeal the rejection and re-write a letter to reviewers. 
We have modified the manuscript and included a point-by-point response to the concerns raised by editor and reviewers below.
I kindly ask you for a re-consideration of publication.
Thank you very much in advance.
Sincerely yours,
Kirsten Ahring
Response to reviewers combined with “Cover letter” (edited version from August 30, 2020)
My colleagues and I humbly re-submit our manuscript entitled “Effect of offering casein glycomacropeptide versus free synthetic amino acids to early treated phenylketonuria mice” for your re-consideration. 
We wish to thank you all for your constructive comments in this second round of review. Your comments provided valuable insights to refine its contents and analysis. In this document, we try to address the issues raised as best as possible
Reviewers answers to PLOS ONE	Authors respond to reviewers PLOS ONE answers	
1. Is the manuscript technically sound, and do the data support the conclusions? 

Reviewer #1: Partly

Reviewer #2: Yes
	Thank you	
2. Has the statistical analysis been performed appropriately and rigorously? 

Reviewer #1: No

Reviewer #2: Yes
 
	(Reviewer #1) Thank you very much for your suggestion regarding using one-way ANOVA instead of multiple t tests.  During my PhD study period, I did seek advice at the Statistical Department at the University of Copenhagen and the teacher recommended that I should use multiple t-tests in order to obtain the most transparent results. He stated, that in his opinion it can confuse the outcome to include to many parameters in the same statistical analysis. Therefore, I choose to keep this method as kindly offered from the Academic Editor. 
	
3. Have the authors made all data underlying the findings in their manuscript fully available?
Reviewer #1: Yes

Reviewer #2: Yes

	Thank you	
4. Is the manuscript presented in an intelligible fashion and written in standard English?
Reviewer #1: Yes

Reviewer #2: Yes
	Thank you	
Comments from Reviewer 1#	Answers from author to Reviewer 1# comments 	
I recommend that the bar graphs be converted to whisker plots so that the true mean and range of the data can be inspected. This would apply to Figure 3 and supplementary Figure 1.
	We have now changed the paper substantially 

Thank you for the suggestion, we have converted the bar graphs Figure 3 and supplementary Figure 1 to whisker plots. 	
For Figure 3, I recommend that the amino acids be separated from the neurotransmitters into different charts as the amounts measured differ by two orders of magnitude and it makes inspection of the current figure difficult.	Thank you for the recommendation for Figure 3, we have separated the amino acids from the neurotransmitters into different charts (different y-axis) to make the inspection of the current figure easier.
	
If it's possible to find a suitable short phrase or abbreviation, I recommend naming the actual treatment of the groups in the figures rather than using Group 1, group 2, etc in the figures as I found myself having to go back repeatedly to refresh my memory on what each treatment group actually was. I can remember a couple treatments but not five.
	We do agree that a suitable short phrase or abbreviation would be preferable for naming the actual treatment of the groups. We have done as follows: Re-named the 5 groups: G1(N-CGMP), G2 (N-CGMP-LNAA), G3 (N), G4 (CGMP-EAA-LP) and G5 (FSAA-LP). 	
As the authors have clearly learned, the lack of either wild type B6 mice or untreated B6-Pahenu2 mice as controls in the behavioral studies make the current results difficult to interpret. Given the decrease in distance traveled, the animals are clearly learning the maze, which I suspect is an improvement over what they would have done had they not been treated from early in life, but do the authors have any maze data on wild type mice or untreated B6-Pahenu2 mice (not collected contemporaneously obviously) to compare to?
	Thank you for addressing this very important issue regarding lack of wild type mice. We did not have a wild type mouse to compare to, which we initial found was not a problem, since the main objective of the study was to determine the differences between the diet compositions. We are aware that similar studies has used wild type mouse to compare with ( (Ney, 2008) (Vliet, 2016) and we have realized afterwards, that this could have been useful for the Barnes maze study, since there were no differences between the groups, despite the different diet regimes. We have included a comment about this in the discussion section.	
The timing of the dietary treatment is bit unclear from the manuscript. It is stated in the manuscript that the pups were products of homozygous Pahenu2 dams treated with Phe-restricted diet and this diet was continued through weaning. Figure 1 shows experimental diets being initiated sometime between week 4-16 and continuing to week 19. When precisely did the experimental diet start? What diet were the mice fed between weaning and the onset of the experimental diet. This would have influenced their ability to perform the maze testing.
	The experimental diet started at week 4, right after weaning was ended. The breeding animals were maintained on Phe-free semi synthetic diet (Harlan Laboratories), also during pregnancy and weaning period, a Maternal PKU diet. As the diet was free of Phe, the drinking water was supplemented with Phe (Sigma-Aldrich Chemie) to a final concentration of 62.5 mg/ml during that period.
We have now included a new figure 1 to make it more clear.	
Incidentally, the use of the low Phe diet in the dams clearly allows them to generate progeny but since their milk would contain normal concentrations of lactalbumin, I would except Pahenu2 homozygous pups to become hyperphenylalaninemic regardless of whether the dam continued on a low Phe diet or not. Do the authors have blood Phe data on the progeny at weaning or prior to the institution of the experimental diets? The pups may have suffered sufficient brain damage from hyperphenylalaninemia during the juvenile period that Phe lowering treatment instituted later in life may have had little effect on behavior in the animals.

	Yes we agree that this could be a problem however It was not possible to take a blood test for determination of the total amino acid profile at week 1 of diet intervention since the mice at that time, were too small to tolerate blood sampling, but as all animals were treated equal before week 1, we assume that the start value was similar in all groups. This is included in the paper.
	
The current concept of LNAA transport at the blood brain barrier being mediated solely via the LAT-1 transporter is exceedingly inadequate. There is evidence for a number of other transporters, some that transport amino acids in reverse direction against the gradient, being involved in brain amino acid homeostasis. The existence of this system is why dietary manipulation in this experiment had little effect upon brain amino acid content other than Phe.

The authors make the statement in their introduction that the imbalance in brain LNAA is 'probably the primary cause of disrupted brain development in this disorder' and then cite a single reference. This statement denies abundant evidence and dozens of other publications on a multitude of other potential pathogenic mechanisms; the statement should be eliminated
	We have removed the sentence as requested by reviewer.	
The authors find improved bone density primarily in the groups of animals on the Phe-restricted diets yet make no statement about the potential pathogenesis of hyperphenylalaninemia itself upon bone health.	Thank you for pointing out that statement about the potential pathogenesis of hyperphenylalaninemia upon bone health was missing. We have in the discussion included the impact on bone density. 
	
The authors conclude that CGMP can be a 'relevant supplement for the treatment of PKU.' I don't disagree. However, in my opinion, the more important conclusion is that dietary Phe-restriction, potentially using CGMP as part of the Phe-restricted diet, leads to vastly superior outcomes using multiple measures in comparison to ignoring Phe restriction and trying to supplement with LNAA	Also thank you for emphasizing, that it is important to highlight the fact, that taking large amounts of large neutral amino acids orally can help protect the brain from damage from elevated phenylalanine, but the main lowering effect on brain phenylalanine comes from lowering the blood phenylalanine. We thought we already indicated that but have now expanded the discussion section where we describe in more detail with the work of others, as you advised us to do.	
Comments from Reviewer 2#	Answers from author to Reviewer 2# comments 	
This is a valuable study, but the separation of Phe intake through water from all other AA in G4 and G5 makes it difficult to compare data. The text is very dry to read and I would recommend to combine results and discussion to give the reader more guidance. Some of the discussion is just a repeat of results. Please also include the supplementary data in the main part of the manuscript.
	We have now changed the paper substantially 


Thank you for your valuable advices regarding design of the manuscript. We do agree that the text can be dry to read, and therefore we have combined “results-“and “discussion sections” a bit more as suggested and included the supplementary data in the main part of the manuscript. 
	
Page 13 diets: Please explain diet composition of G3.
	The composition of G3 is a complete diet, consisting of casein (MIPRODAN^®^ 30), sucrose, corn starch, corn oil, cellulose, mineral- and vitamin-mix. Basically, the nutritional value is the same as the other experimental groups. We have now included a detailed description of G3 in Table 2
	
Page 13 of submitted pdf; Phenylalanine supplementation: 57mg/ml would be an extraordinary amount of Phe (345 mM) and not suitable for the mouse strain. Probably 57mg/L.
	Thank you for pointing out that 57mg/ml of Phenylalanine supplementation would be an extraordinary amount of Phe (345 mM) and not suitable for the mouse strain.  In fact, 25 mg/ml was used to produce the drinking mixture, where 9 ml (225 mg) was added to 391 ml of water, which provided a final concentration of 563 mg/l	
Page 19/20: There are two error messages in the text.
	Thank you for making us aware of this, it is corrected now.	
Table 2: Threonine is elevated in G4 and G5. It is an essential AA and diet 5 has a significant amount, but diet 4 doesn't seem to have Thr. Please explain. Arginine is consistent and found in G4 and G5 diets.	We are glad that you pointed this out, since it is not clear at all from the table 2, that the CGMP product contains large amount of Thr. We have now added the data from CGMP-20 to make it more obvious	
I would recommend to convert table 2 into a bar graph.
Figures in the pdf are of unacceptable resolution.	We hope the resolution of the figures have improved with the new system: uploading figure files to the Preflight Analysis and Conversion Engine (PACE) digital diagnostic tool https://pacev2.apexcovantage.com/	
Fig 3 Please present control data from a background mouse strain. The figure caption needs to explain the information content of the right panel. 
	Unfortunately, the mouse strain we used from AAU had no wild type as described in the introduction section, but we have found useful information from other similar studies (Ney, 2008) (Vliet, 2016)
We were aware from the start of the study, that a wild type of the mouse strain would have been a useful tool. However, the information we needed from the study would be possible to gain, also without a wild type, but that fact determined the design of the study: 2 groups with pure GCMP, 1 group on normal diet, 2 groups with FSAA
We expected to find a difference between the groups due to the fact, that late-diagnosed/untreated PKU patients over the years have been studied and observed.	
Do not abbreviate brain areas.	We have  now changes to full names for brain areas 	

As suggested by you, we have published our protocol, which can be found here (DOI): https://www.protocols.io/view/effect-of-offering-phenylketonuria-pku-mice-glycom-bkg9ktz6
We have removed the phrase “data not shown” in the manuscript and added the data.
We addressed the suggestion of reviewer 2 and integrated the Supporting Information. 
Funding Statement: The research was conducted as part of an lndustrial PhD program, which in Denmark is a three-year industrially focused PhD project where a student (Kirsten Ahring) is hired by a company (Arla Foods lngredients (AFI)) and enrolled at a university (Copenhagen University) at the same time. The industrial
PhD program was managed by Innovation Fund Denmark and partly funded by AFI (https://ufm.dk/en /research-and-inn ovation/funding-proqrammesfor-research-and-innovation/find-danish-funding-programmes/programmes-managed-byinnovation-fund-denmark/industrial-phd ). AFI produces and commercialize the food ingredient; Casein glycomacropeptide which was used in the research.
Competing Interests Statement: Erik Jensen was the industrial supervisor for the above described PhD project, and his mandatory obligation was to contribute with inputs to study design, formulation of test diets and study manuscript according to agreements and ethical standard. However, Erik Jensen did not have the final decision about these issues and this affiliation does not alter our adherence to PLOS ONE policies on sharing data and materials. Erik Jensen has sent a written signed statement regarding this (separate file).
Once again, we thank you for the time you put in reviewing our paper and look forward to meeting your expectations. Since your inputs have been precious, in the eventuality of a publication, we would like to acknowledge your contribution explicitly. 

Sincerely yours,
Kirsten Kiær Ahring
Metabolic nutritionist, M.Sc., Ph.D.
Copenhagen University Hospital
Copenhagen, Denmark
